# Psychiatric Disorders in HTLV-1-Infected Individuals with Bladder Symptoms

**DOI:** 10.1371/journal.pone.0128103

**Published:** 2015-05-27

**Authors:** Glória O. Orge, Thais R. Dellavechia, José Abraão Carneiro-Neto, Lucas Araújo-de-Freitas, Carla H. C. Daltro, Carlos T. Santos, Lucas C. Quarantini

**Affiliations:** 1 Programa de Pós-graduação em Medicina e Saúde, Universidade Federal da Bahia, Salvador, BA, Brasil; 2 Programa de Pós-Graduação em Ciências da Saúde, Universidade Federal da Bahia, Salvador, BA, Brasil; 3 Departamento de Neurociências, Hospital Universitário Professor Edgard Santos, Universidade Federal da Bahia, Salvador, BA, Brasil; 4 Escola de Nutrição, Universidade Federal da Bahia, Salvador, BA, Brasil; 5 Departamento de Ciências Exatas, Universidade Estadual de Feira de Santana, Feira de Santana, BA, Brasil; 6 Instituto de Saúde Coletiva, Universidade Federal da Bahia, Salvador, BA, Brasil; Ecole Normale Supérieure de Lyon, FRANCE

## Abstract

**Background:**

Previous studies have reported high rates of depression and anxiety in HTLV-1 infected individuals with the neurological disease and in the asymptomatic phase. No study has investigated the rates in individuals that already show bladder symptoms without severe neurological changes; that is, during the oligosymptomatic phase. The present study investigated patients in this intermediate form on the spectrum of the infection.

**Methodology/Principal Findings:**

Participants answered a sociodemographic questionnaire, the Mini International Neuropsychiatric Interview Brazilian Version 5.0.0 (MINI PLUS) and the Hospital Anxiety and Depression Scale (HADS). Data analysis was performed in STATA statistical software (version 12.0). Depressive disorder was the most frequent comorbidity. Current depressive disorder was higher in the group of overactive bladder subjects (11.9%), and lifelong depression was more frequent in the HAM/TSP group (35%). The three groups had similar frequencies of anxiety disorders. Increased frequency and severity of anxiety and depression symptoms were observed in the overactive bladder group.

**Conclusion/Significance:**

The results suggest that individuals with overactive bladders need a more thorough assessment from the mental health perspective. These patients remain an understudied group regarding psychiatric comorbidities.

## Introduction

Human T-lymphotropic virus type 1 (HTLV-1) is endemic in many countries in Africa, the Caribbean, South America and Japan [1]. Despite being considered a virus with low morbidity, it produces an infection with a large spectrum of different symptom levels: on one hand, individuals have no symptoms, and on the other hand, individuals may develop HTLV-1 associated myelopathy/tropical spastic paraparesis (HAM/TSP) [2,3] or adult T-cell leukemia/lymphoma (ATLL) [4]. Evidence shows that a large percentage of individuals develop many associated diseases and clinical or neurological symptoms. Thus, complaints of muscle weakness, paresthesia and hyperreflexia in the lower limbs [5,6] erectile dysfunction [7], as well as inflammatory diseases such as uveitis [8], periodontitis [9] and infective dermatitis [10] have been described.

HTLV-1 infected individuals also present increased urinary frequency, urgency, incontinence [11] and sometimes a neurogenic bladder, presenting urodynamic parameters similar to those individuals with myelopathy, raising the hypothesis that it is a oligosymptomatic form of HAM/TSP [12] Even individuals newly diagnosed by blood banks have a significantly higher frequency of neurological, ocular and rheumatic complaints than negative controls [13].

Psychiatric comorbidities have been considered a pertinent topic, as they are associated with clinical problems commonly described in viral infections. By far the best characterized associations are related to the human immunodeficiency virus (HIV) and human hepatitis C (HCV) studies [14], mainly describing the presence of depressive and anxious symptoms which are not always diagnosed, and aspects of quality of life [15–19]. The relationship between HTLV-1 infection and the presence of psychiatric disorders has received little investigation; however, the existing data suggests that individuals infected with this virus have a high frequency of depressive and anxious symptoms, resulting in a perception of impaired quality of life [20–25]. The clinical diagnosis of various diseases has been reported in the literature as a stressful and/or traumatic experience in itself [26,27]. Blood donor candidates, when notified of HTLV-1 infection, develop a high degree of distress that produces negative psychological and social effects [28].

Two studies conducted in blood banks presented conflicting results related to the prevalence of depression and anxiety disorders: One in Brazil found a frequency of 39% for depressive disorder among blood donors with positive serology to HTLV-1, while negative donors showed 8% [25]. The other study was conducted in the USA and presented a small increase in the prevalence of depression among blood donors with positive serology to HTLV-1, compared to seronegative patients: 5.4% and 2.1% respectively; the difference was not significant after adjusting for confounding variables. The same result occurred in relation to generalized anxiety disorder (GAD), where the prevalence was 5.4% in seropositive and 2.6% in seronegative patients [23]. After diagnosis, these effects may remain; as shown in two clinical studies conducted in Northeastern Brazil involving asymptomatic and HAM/TSP outpatients infected with HTLV-1, which described a 34% prevalence of depression among these subjects [20,21].

It is worth noting that there is an intermediate form of this infection, constituting a group of mildly symptomatic patients that already show symptoms without severe neurological changes [12]. Previous studies did not investigate these individuals, which may lead to the misinterpretation that they present reduced psychological distress when compared with HAM/TSP subjects. Consequently, identifying this group's behavioral profile and comparing them with asymptomatic and HAM/TSP individuals having a similar time of diagnosis for HTLV-1 may lead to a better understanding of whether this stage of the disease has peculiarities that deserve special attention. To our knowledge, there is no previous study assessing psychiatric disorders in HTLV-1 oligosymptomatic individuals.

The aim of this study is to evaluate the frequency of psychiatric disorders in HTLV-1 infected individuals in the following clinical forms: asymptomatic, overactive bladder (OAB) and HAM/TSP, who were treated in a multidisciplinary outpatient HTLV clinic in a referral center in Northeastern Brazil.

## Materials and Methods

### Study Design

This is an observational cross-sectional study of 172 HTLV-1 infected individuals treated in the multidisciplinary outpatient clinic at the Complexo Hospitalar Professor Edgard Santos at the Federal University of Bahia (UFBA), from January 2012 to April 2014.

### Subjects

During the study period, a convenience sample of 172 of HTLV-1 infected individuals was selected consecutively divided in three groups: 40 with HAM/TSP, 42 with isolated urinary symptoms and 90 asymptomatic. The number of subjects per group was in the same proportion compared to the cohort study ongoing since 2004 with 414 individuals followed in the HTLV-1 clinic at Hospital Universitário Prof. Edgard Santos, Salvador, Bahia, Brazil. Subjects are evaluated at 6 to 12 month intervals by a multidisciplinary team consisting of neurologists, urologists, rheumatologists, psychologists and dentists. A standardized assessment is conducted each year with questions about clinical, neurological and urological symptoms. Physical and neurologic exams are also performed.

### Inclusion Criteria

Individuals enrolled in this study were older than 18 years, with serological diagnosis performed by ELISA (Cambridge Biotech Corp., Worcester, MA, USA) and confirmed by Western Blot (HTLV blot 2.4, Genelabs, Singapore). Inclusion in each group was made by the neurologist's classification according to the neurological scales: Expanded Disability Status Scale (EDSS) [29] and Osame's Motor Disability Score (OMDS) [30]. Patients were considered asymptomatic when the EDSS = 0 and Osame = 0; oligosymptomatics: patients with complaints of overactive bladder (OAB) with EDSS ≥ 1 and Osame = 0, clinicaly confirmed by a urologist; and HAM/TSP: those with EDSS ≥ 2 and/or Osame> 1.

### Exclusion Criteria

HTLV-2 carriers and individuals with indeterminate Western Blot results were not included in this study.

### Instruments for data collection

All participants answered a sociodemographic and clinical questionnaire. The outcome measures were assessed by the Mini International Neuropsychiatric Interview Brazilian Version 5.0.0 (MINI PLUS), which includes questions about suicide risk. Low risk is characterized by thoughts of death; medium risk characterized by ideation or planning of suicide; and high risk characterized by attempt of suicide [31] Finally, the intensity of anxiety and depressive symptoms was assessed throughout the Hospital Anxiety and Depression Scale (HADS), a self-report scale measure [32].

### Data Analysis

Fisher's exact test was used for categorical variables and the Kruskall-Wallis test for continuous variables. Univariate logistic regression was used for dichotomic outcomes and Polytomous Logistic Regression was used to calculate odds ratio and respective 95% confidence intervals to describe the association between psychiatric disorders and the 3 clinical conditions of HTLV infection. All variables identified by the likelihood ratio with p-value≤ 0.200 were entered in the multivariate model to adjust for potential confounders. A p-value < 0.05 was used to determine statistical significance. Data was analyzed using the STATA statistical software (version 12.0).

### Sample size calculation

The sample size was calculated considering 8% prevalence of the event based on a pilot study. The desired precision was 5%, the alpha error 5% and a power of 80%. The result estimated was 95 individuals.

### Ethical considerations

This study was conducted in accordance with the tenets of the Declaration of Helsinki. This study was approved by the Ethics Committee of the Climério de Oliveira Maternity Ward, Federal University of Bahia/UFBa, Brazil No. 260/2009. All participants signed the Informed Consent Form (ICF) prior to the collection of data.

## Results

A total of 172 HTLV-1 infected subjects were included in the study. Ninety individuals (52%) were asymptomatic, 42 (24%) had overactive bladder and 40 (23%) had HAM/TSP.

The sociodemographic characteristics of the participants are described in Table 1. The variables: gender, age, marital status, education, time since diagnosis, and comorbidities were distributed similarly between the three groups.

**Table 1:** Sociodemographic and clinical characteristics of HTLV-1 infected individuals treated at the multidisciplinary outpatient clinic at Complexo Hospitalar Professor Edgard Santos/UFBA—Salvador/BA. 2014.

Variable		Asymptomatic Carriers	Overactive Bladder	HAM/TSP	P*
		N = 90	N = 42	N = 40	
		(%)	(%)	(%)	
Gender					0.077
	Female	61(67.8)	33(78.6)	22(55.0)	
	Male	29(32.2)	9(21.4)	18(45.0)	
Median age (years)		52.5	54.5	61.5	0.236
Age **					0.124
	< 53 years old	49(54.4)	20(47.6)	14(35.0)	
	≥ 53 years old	41(45.6)	22(52.4)	26(65.0)	
Marital status					0.236
	Married	59(65.6)	24(57.1)	20(50.0)	
	Not married	31(34.4)	18(42.9)	20(50.0)	
Education					0.558
	< 8 years	49(54.4)	27(64.3)	22(55.0)	
	≥ 8 years	41(45.6)	15(35.7)	18(45.0)	
Income ***					0.297
	≤ 2 minimum wages	72(80.0)	38(90.5)	32(80.0)	
	> 2 minimum wages	18(20.0)	4(9.5)	8(20.0)	
Ethnicity					0.109
	White	7(7.8)	10(23.8)	4(10.0)	
	Non White	83(92.2)	32(76.2)	36(90.0)	
Time of diagnosis					0.637
	< 8 years	44(48.9)	17(40.5)	17(42.5)	
	≥ 8 years	46(51.1)	25(59.5)	23(57.5)	
Clinical comorbities****		28(31.4)	23(54.8)	16(40.0)	0.085

*Fisher´s exact test

** Dichotomized by median split

*** A minimum wage about US$ 330

****Hipertension and diabetes

It was observed that there was a predominance of women, about 60%, more pronounced in the overactive bladder group (78.6%) followed by the asymptomatic group (67.8%) and HAM/TSP group (55%). However, no statistically significant difference was observed.

The median age was 52.5 in the asymptomatic group, 54.5 in the overactive bladder group and 61.5 in the HAM/TSP group. With regard to age, it was noticed that most asymptomatic individuals were less than 53 years old (54.4%). The majority of individuals in the two symptomatic groups were older than 53 years (52% in the overactive bladder group, and 65% in patients with HAM/TSP). Most individuals in the three groups were married or had a stable relationship and had less than eight years of formal education.

Regarding monthly income and ethnicity, it was observed that all groups were similar: 80–90% had a monthly income of less than two multiples of the minimum wage, and 76–92% were Afro-American. A slight majority had more than eight years since diagnosis. Regarding the presence of clinical comorbidities, hypertension and diabetes, the greatest percentage was found in the overactive bladder group, but it had no statistical significance.

The distribution of the frequencies of psychiatric disorders diagnosed in different groups of individuals infected with HTLV-1: asymptomatic, individuals with overactive bladder and HAM/TSP is described in Table 2.

**Table 2:** Frequency and Multivariate Analysis of psychiatric disorders related to the clinical conditions of HTLV-1 infected individuals from the multidisciplinary outpatients clinic at Complexo Hospitalar Professor Edgard Santos/UFBA—Salvador/BA. 2014.

Variable		Assymptomatic Carriers	Overactive Bladder	HAM/TSP	aOR (IC95%)* Overactive bladder	aOR (IC95%)* HAM/TSP
		N = 90	N = 42	N = 40		
Psychiatric disorders						
	Current depression	4.4%	11.9%	10.0%	2.9(0.74–11.43)	2.38(0.57–10.07)
	Lifelong depression^a^	17.8%	31.0%	35.0%	1.93(0.82–4.55)	2.85(1.19–6.80)**
	Anxiety disorders	11.1%	9.5%	10.0%	0.84(0.25–2.85)	0.89(0.26–3.02)
	Other disorders ^b^	13.3%	19.0%	15.0%	1.94(0.69–5.41)	1.17(0.39–3.55)
Risk of suicide						
	Low ^c^	20.0%	19.0%	15.0%	1.28(0.48–3.43)	1.08(0.37–3.17)
	Moderate/ High ^c^	5.6%	16.7%	15.0%	4.30(1.19–15.59)	4.49(1.17–17.30)
Median*** HAD anxiety (Q1-Q3) ^d^		3(1–6)	7.5(3–13)	4 (1–8)	4.03(2.21–5.85)**	1.61(0.07–3.15)**
Median*** HAD depression (Q1-Q3)^e^		3(1–6)	6(3–10)	4 (2–9)	2.89(1.24–4.54)**	1.3(0.48–3.58)**

*Adjusted ODDS ratio

^a^ = adjusted by gender

^b^ = adjusted by gender and age

^c^ = adjusted by age

^d^ = adjusted by gender, age and ethnicity

^e^ = adjusted by age and clinical comorbidities.

Other disorders = alcoholism, eating disorders and psychosis

** ≤ 0, 05

*** Kruskall-Wallis Test.

We observed a higher frequency of current depressive disorder in the group of overactive bladder subjects (11.9%), corresponding to almost three times greater likelihood of presenting this disorder when compared to the asymptomatic group (4.4%). The group of patients with HAM/TSP was twice as likely to develop this disorder than asymptomatics (10% x 4.4%, respectively), although this difference does not show statistical significance.

Lifelong depression was most frequent in the HAM/TSP group (35%), about two times more prevalent when compared to the asymptomatic group, with statistical significance that remained even after adjusting for confounding variables. Next came the overactive bladder group (31%), despite there being no statistical difference between the two symptomatic groups.

The symptoms assessed by the Hospital Anxiety and Depression Scale (HADS) regarding the depression sub-scale scores indicated an increased severity of depressive symptoms in subjects in the overactive bladder group. There was a median of 3 in the group of asymptomatic carriers, 6 in the group of subjects with overactive bladder, and 4 in the group of HAM/TSP (p < 0.001).

Anxiety disorders: panic disorder, agoraphobia, social phobia, generalized anxiety disorder (GAD) and obsessive-compulsive disorder (OCD) were grouped as a variable called anxiety disorder, with very similar frequencies between the three groups: asymptomatic carriers 11.1%; individuals with overactive bladder 9.5% and patients with HAM/TSP 10%.

The symptoms assessed by the Hospital Anxiety and Depression Scale (HADS) indicate an increased severity of anxiety symptoms in subjects in the overactive bladder group compared to the asymptomatic group (p < 0.004). The score on the anxiety subscale showed a median of 3 in the group of asymptomatic carriers, 7.5 in the group of overactive bladder subjects, and a median of 4 in the group of HAM/TSP.

Even after controlling for confounding variables in multivariable analysis, symptoms of anxiety and depression (assessed by HADS) remained significantly higher in the oligosymptomatic group when compared to the asymptomatic group.

Other chronic disorders—alcoholism, eating disorders and psychosis—were diagnosed primarily among patients with overactive bladder (19%) occurring twice as often as the asymptomatic group, although this difference did not show statistical significance.

Regarding the risk of suicide, the frequency was similar between the three groups, although it was higher in the overactive bladder group (35.7%), followed by HAM/TSP (30%); the risk being lowest in the asymptomatic group (25.6%). Although no statistical significance was found, it was observed that this risk was three times as common among individuals with overactive bladder in comparison to the asymptomatic group (16.7% x 5.6% respectively).

The variables included in the multivariable model were: gender; age; ethnicity and clinical comorbidities.

## Discussion

The results of this study demonstrate that HTLV-1 oligosymptomatic subjects are just as affected by psychiatric comorbidities as the HAM/TSP individuals. To this date, the few studies describing the frequency of psychiatric disorders among HTLV-1 were conducted among asymptomatic or HAM/TSP patients [20–25]. According to our knowledge, this is the first study to investigate psychiatric disorders that specifically addresses individuals who have symptoms of overactive bladder, which represents an early stage of neurological disease.

Depression was the most frequent comorbidity in this study and its frequency among all individuals infected with HTLV-1 in our sample was similar to those found in earlier studies [20,21,25] and was higher than the prevalence of 2–10% found in the general population of three Brazilian cities [33]. We emphasize that our findings are also in accordance with the literature addressing other chronic diseases, which describes 5–10% of depressive comorbidity in primary health care, 10–14% among outpatients and 15–31% among patients with chronic diseases [34] such as diabetes [35], coronary heart disease [36], HIV [37], and HCV [15,38].

Different hypotheses attempt to explain the prevalence of depression in HTLV-1 infected individuals. Some authors suggest that the virus may be related to the pathogenesis of depression inducing pro-inflammatory cytokines production such as IL-1, TNF-alpha and IL-6 [20,21]. On the other hand, Guiltinan et al. believe in a stronger association with cultural and socioeconomic aspects, since his study identified lesser prevalence than those found in developing countries like Brazil [23]. The differences between the prevalence in Brazilian and the North American study are probably due to a combination of several factors: there was no uniformity of the instruments used for diagnosis, different genetic background of individuals, different socio-demographic characteristics and different origins of participants: from specialized clinics or blood banks [20–25] We agree with Guiltinan, 2013, that the socioeconomic aspects are more relevant as income and education in the American sample were significantly higher than those of participants in Brazilian studies. This hypothesis is plausible according to the findings of a meta-analysis which showed that socioeconomic disparities are strongly associated with major depression [39].

It is noteworthy that depressive disorder was more frequent among symptomatic individuals, corresponding to being about twice as likely to have depression compared to the group of asymptomatic carriers. Notable as well is the suicide risk. Although we emphasize that most of the subjects presented low risk (characterized by thoughts of death that do not translate into ideation, planning or attempt), it is important to notice that there were individuals who had moderate/high risk. Risk of suicide was three times higher in the two symptomatic groups compared to asymptomatic, with slightly more frequency in the overactive bladder group. It is known that chronic and severe diseases impose losses and limitations that may constitute an important risk factor for suicide, especially in the presence of comorbid depression and anxiety [40,41].

Psychiatric disorders may remain unnoticed due to the fear of stigma that still exists in this area; individuals do not report their symptoms if they are not asked about them. Thus, the manifestation of depressive disorder may remain underdiagnosed by not being investigated. This phenomenon seems to occur in various pathologies: chronic renal disease [42], diabetes mellitus [43], HCV [16], leprosy [44] etc.

The prevalence of anxiety disorders was similar among the three groups and also similar to the prevalence of 9–18% of the general population in three Brazilian cities [33]. However, the scores of the HADS sub-scale of anxiety and depression that measure the severity of symptoms were more pronounced in the overactive bladder group, even in the absence of a confirmed diagnosis of the anxiety disorder. We hypothesize that it could be secondary to the expectancies generated by new symptoms and a non-defined prognosis. Our findings are in accordance with previous studies that describe OAB symptoms as bothersome, with an impact on emotional well-being, increasing anxiety and depression symptoms, and negatively affecting the quality of life [45–47].

The prevalence of depression and anxiety found by Souza et al [24] and Gascón et al [22], who investigated through severity scales, was higher than our findings. Such differences seem to be due to the fact that we used the HADS scale, which prioritizes non-somatic symptoms. They used, respectively: the Hamilton Rating Scale for Depression and the Beck Depression Inventory (BDI), which also consider them. Some symptoms such as fatigue, anorexia and insomnia may be related to the HTLV-1 itself, but can also be interpreted as related to major depression, and consequently inflate the total scores of depression.

The findings of the present study should be interpreted while taking into account their methodological limitations. Firstly, the evaluation of subjects monitored in a specialized clinic at tertiary level limits its generalization to other populations. Secondly, due to the cross-sectional nature of the study, it is not possible to determine the causal relation between depression/anxiety and clinical forms of the HTLV-1 infection. Thirdly, convenience samples may not provide an ideal randomized sample; the most severe and asymptomatic subjects could be the most absent. Finally, we cannot measure how much comorbid psychiatric disorders modify patient’s attendance at the outpatient HTLV clinic.

In conclusion, the overall results suggest that symptomatic individuals infected with HTLV-1, and especially those with symptoms of overactive bladder, constitute a group that need a more thorough assessment from the mental health perspective, so they may cope with this chronic disease that can lead to disability. This fact, together with a scenario of unfavorable social factors, may contribute to the onset of depression and anxiety comorbidities. Primary health caregivers should be aware of these conditions in order to diagnose and intervene appropriately, since depression can lead to increased somatic complaints without identifying biological causes, which can lead to an increased use of health services and, therefore, rising costs.

Further studies regarding the influence of biological factors on the pathophysiology of depression are also required of HTLV-infected subjects, such as pro-viral load and pro-inflammatory cytokines production and their association with depression. Furthermore, a comparison between HTLV-1 infected and uninfected individuals should also contribute to this subject.
